# Acceptability of early infant male circumcision among chinese parents: strategy implications of HIV prevention for china

**DOI:** 10.1186/1471-2458-12-738

**Published:** 2012-09-04

**Authors:** Lianjun Pan, Aixia Zhang, Rong Shen, Zhong Wang

**Affiliations:** 1 State key Laboratory of Reproductive Medicine, Department of Urology, Nanjing Maternity and Child Health Care Hospital Affiliated to Nanjing Medical University, NO.123 Tianfeixiang, Mochoulu, Nanjing City, 210004, China; 2 Department of Obstetrics and Gynecology, Nanjing Maternity and Child Health Care Hospital Affiliated to Nanjing Medical University, Nanjing, 210029, China; 3 Department of Urology, Shanghai Ninth People’s Hospital Affiliated to Shanghai Jiao Tong University School of Medicine, Shanghai, 200011, China

**Keywords:** Early infant male circumcision, Chinese, AIDS, Prevention

## Abstract

**Background:**

Recent evidence has confirmed that circumcision can be performed as a preventive strategy for HIV and early infant male circumcision (EIMC) is regarded to be safer than circumcision in adulthood; however, limited data are available in the literature about EIMC in China. Therefore, the present study was designed to determine the willingness and attitudes of Chinese parents on newborn male circumcision so as to provide data for exploring the feasibility of implementing EIMC as an HIV prevention strategy in China.

**Methods:**

Simple random sampling was used to draw participants from parents who had a newborn son delivered at Nanjing Maternity and Child Health Care Hospital, which is affiliated to Nanjing Medical University, between March and December 2010. A questionnaire was used to determine general medical knowledge or information about circumcision, attitudes about EIMC, and level of decision-making on circumcision for the newborn son.

**Results:**

Data derived from 558 responses were analyzed and the ratio of respondents was 56.3% for fathers and 43.6% for mothers. Of the respondents, 34.4% agreed to circumcise their newborn son, and the level of agreement was 3.25 ± 1.17 (range, 1–5 with “1” being “reluctantly agree” and “5” being “very strongly agree”). The major reason for EIMC was for health (44.8%), followed by doctor’s advice (31.2%). The major reason not to agree to EIMC was concern about pain (50.5%), followed by the risk of the procedure (23.5%).

**Conclusion:**

The willingness and acceptability of EIMC in China is low and the parents of newborn sons are usually not very affirmative when making a decision on such a procedure, suggesting that significant effort will be needed if EIMC is to be implemented as an HIV prevention strategy for China.

## Background

The results from three randomized controlled clinical trials conducted in Africa confirmed that male circumcision (MC) lowers the risk of HIV acquisition by 55%-76% in heterosexual men [1-3]. Based on the evidence, the WHO/UNAIDS has recommended a rapid implementation of MC in countries with a high prevalence of HIV to maximize intervention effectiveness at the population level [4]. To ensure that this protective effect is sustained the WHO/UNAIDS also recommend early infant male circumcision (EIMC) [4]. Boys circumcised during the first month of life have significantly lower frequencies of urinary tract infections and bacteremia compared with uncircumcised boys, and serious complications are rare [5,6]. Furthermore, EIMC is also considered to be simpler and safer than circumcision in adulthood, with a lower risk for surgical errors, infections, and other adverse effects [7]. Reports from some Africa countries have also provided evidence showing that EIMC has high acceptability for HIV/AIDS prevention [8-10].

Although the prevalence of HIV/AIDS in China is currently low, the annual morbidity and mortality rates have exhibited upward tendencies since 2003, and the HIV/AIDS epidemic is spreading from high-risk groups to the general population as a result of sexual transmission [11,12]. In recent years, the proportion of reported HIV/AIDS cases in China accounted for by homosexual and heterosexual transmission has increased year after year. The proportion of cases of HIV resulting from sexual transmission increased from 33.1% in 2006 to 76.3% in 2011, and the fraction arising from heterosexual transmission rose from 30.6% in 2006 to 53.8% in 2010 [11]. Because preventing HIV infection is critical to control and ultimately bring an end to the HIV/AIDS epidemic, effective and more accessible intervention strategies, such as EIMC, are urgently needed in China. Additionally, more widespread use of MC could significantly improve the cervical cancer rate due to persistent high-risk HPV infections, a major health problem in Chinese women because evidence has shown that MC can protect against HPV infections and related diseases in men and women [13]. However, only 2.66% of men are circumcised in China usually because of a tight foreskin; circumcision is generally performed in adulthood or childhood, but not at the time of birth [14]. The willingness and acceptability of EIMC among Chinese parents is unknown given a lack of history and cultural norms endorsing neonatal and adult circumcision. Therefore, the present study was designed to determine the willingness and attitudes of Chinese parents on circumcision in newborn males so as to provide data for exploring the feasibility of implementing EIMC as an HIV prevention strategy in China.

## Methods

This study was conducted at the Nanjing Maternity and Child Health Care Hospital, which is affiliated to the Nanjing Medical University, between March and December in 2010 and approved by the Ethics Committee of Nanjing Maternity and Child Health Care Hospital (FYLL1012). The study participants were parents who had a newborn son delivered at the hospital. The inclusion criteria were Chinese ethnicity and basic Chinese literacy. The participants who were foreigners, or Chinese who declined to participate in the study, or Chinese who could not complete the questionnaire were excluded. A simple random sampling method was used to draw participants, i.e., by drawing a lot from an opaque bag to decide whether or not the participant was to be interviewed. Informed written consent was obtained from the participants after explaining that the investigation involved circumcision and health. The survey was anonymous and self-administered. The questionnaire items were developed from general medical knowledge or information about circumcision, attitudes regarding EIMC, and the level of decision-making on circumcision for the newborn son. Questions were presented in a multiple choice or yes/no format. Other variables included age, level of education, socio-economic status, religion, and the relationship with the newborn son (mother or father), as shown in Table 1.

**Table 1:** Respondents’ demographics (n = 558)

**Characteristic**	
Age of the respondent (years)	
Father	30.6 ± 3.6
Mother	29.4 ± 3.4
Family income per year*	n (%)
< 40000 RMB (6250 USD)	154 (27.6)
40000-90000 RMB (6250–14062 USD)	298 (53.4)
>90000 RMB (14062 USD)	106 (19.0)
Religion background	
None	506 (90.7)
Buddhism	40 (7.2)
Christianity	10 (1.8)
Islam	2 (0.4)
Relationship between the respondent and neonate	
Father	314 (56.3)
Mother	244 (43.8)
Educational degree (mother)	
Primary or junior high school only	38 (6.8)
High school	80 (14.3)
College or university	382 (68.5)
Masters or greater education	58 (10.4)
Educational degree (father)	
Primary or junior high school only	30 (5.4)
High school	78(14.0)
College or university	370(66.3)
Masters or greater education	80(14.3)

* The median annual income per person in 2011 in China was 19118 RMB (2987 USD).

Preliminary pre-testing was carried out on a group of 30 parents to ensure that the questionnaire items were valid and could be understood. Some of the question items were modified by the feedback obtained. The layout of the print and the arrangement of the questionnaire were designed to be easily read and answered by the participants. Statistical analysis was performed using SPSS (version 17.0; SPSS Inc., Chicago, IL, USA). Descriptive ratings were used for individual questionnaire items, except for the level of agreement regarding circumcision, which was presented as the mean + standard deviation. The chi-square test and Pearson’s correlation coefficient were used to evaluate the relationship among variables. A P value < 0.05 was considered statistically significant.

## Results

A total of 2865 subjects were asked to participate in the current study; 435 declined and 2430 agreed to participate. A sample size of 810 was drawn from the population of 2430, based on a 1:3 sampling ratio, which accounts for about 5% of male neonates in Nanjing city per year. All incomplete (201) and unacceptable responses (51) were excluded. Of the participants, 52.1% were fathers and 47.9% were mothers, with average ages of 29.3 ± 4.1 and 27.8 ± 3.9 years, respectively. The majority of participants (94.2%) had no religious preference. Finally, the data derived from 558 responses were analyzed, with a response rate of 68.9% (558/810). The ratio of the respondents in the final analysis was 56.3% for fathers and 43.6% for mothers, with average ages of 30.6 ± 3.6 and 29.4 ± 3.4 years, respectively. For all the respondents in the final analysis, 90.2% had no religious preference, and most of the participants had a college or higher education. The characteristics of the respondents are shown in Table 1. A majority of respondents considered MC to have health benefits (91.0%), as dangerous (22.9%), to be painful (43.4%), and having potential to enhance sexual function (58.4%) (Table 2).

**Table 2:** Results of the survey (n= 558)

**Questions**	**Response, n (%)**
What do you think about circumcision? (multiple choice)	
Health beneficial	508 (91.0)
Dangerous	128 (22.9)
Painful	242 (43.4)
Enhances sexual function	326 (58.4)
Necessary	190 (34.1)
Do you agree to EIMC?	
Yes	192 (34.4)
No	366 (65.6)
If agreeing to EIMC, who has the final say?	
Father	128 (66.7)
Mather	56 (29.2)
Others	8 (4.1)
Reasons for agreeing to EIMC	
Religion	2 (1.0)
Mother’s choice	12 (6.3)
Father’s choice	11 (5.7)
Doctor’s advice	61 (31.8)
For health	105 (54.7)
Enhances sexual function	1 (0.5)
Reasons for not agreeing to EIMC	
Pain	185 (50.5)
Risky	86 (23.5)
Bad cosmetics	41 (11.2)
Other	54 (14.8)
Have you had ever considered circumcising your son in the future?	
Yes	200 (35.8)
No	358 (64.2)
Best age for childhood circumcision	
Neonatal period	14 (7.0)
1 month −1 year	52 (26.0)
1-3 years	40 (20.0)
3-5 years	26 (13.0)
> 5 years	68 (34.0)
Are you in agreement regarding EIMC?	
Yes	338 (60.6)
No	50 (9.0)
One parent was not involved	170 (30.5)
Where did you get information on circumcision?	
Medical workers	228 (40.8)
Online or other media	194 (34.7)
Others	136 (24.5)
Have you had ever consulted medical workers for your son’s circumcision?	
Yes	124 (22.2)
No	434 (77.8)
Do you think that medical workers have given you sufficient information regarding circumcision?	
Yes	178 (31.9)
No	380 (68.1)
Do the medical workers respect your decision regarding circumcision?	
Yes	500 (89.6)
No	58 (10.4)
Do you desire information on the effect of circumcision on health?	
Yes	470 (84.2)
No	88 (15.8)

Only 34.4% of the respondents agreed to EIMC, and the level of agreement was 3.25 ± 1.17 (range, 1–5 with “1” being “reluctantly agree” and “5” being “very strongly agree”). A significantly higher proportion of fathers compared to mothers agreed to circumcise their newborn sons (40.8% vs. 26.2%; *χ*^2^ = 12.853, P = 0.008). For those who agreed to EIMC, most (66.7%) regarded the father to have the final say. Health, doctor’s advice, and religion were regarded as the major reasons for EIMC in 54.8%, 31.2%, and 1.1% of the parents, respectively. For those who did not agree to EIMC, the major reason was concern about pain (50.5%), followed by the risk of the procedure (23.5%). Most parents (60.6%) were in agreement regarding circumcision of their newborn son. The family income and the level of education of the parents were not associated with agreement regarding EIMC (P = 0.068 for family income; P = 0.064 for mother’s education, and P = 0.334 for father’s education). We also observed that only 35.8% of the parents considered circumcision for their sons in the future; 7% of the parents thought the best time for circumcision was during the neonatal period, and most of the parents (74.0%) believed that the best age for the procedure was after 1 year of age.

When investigating the information on circumcision, 40.8% of those surveyed claimed that they received such information from medical workers and 34.7% through online sources or other media. Most of the respondents (84.2%) desired to know the effects of circumcision on health. Unfortunately, 68.1% of the subjects considered that the medical workers did not provide them with enough information regarding circumcision. All of the results are summarized in Table 2.

## Discussion

Current scientific evidence has convincingly shown that MC is associated with enormous public health benefits, including protection from sexually transmitted HIV, HPV, syphilis, and chancroid, and the penile and prostate cancer rate [15]. Studies of EIMC in countries where it is commonly practiced have consistently shown very low complication rates (< 1%), and a recent review of circumcision complications in pediatric patients concluded that the procedure was safest when performed within the first month of life [16,17]. Therefore, infancy is considered to be the ideal time to perform a circumcision. However, being remarkably different from Western Europe, North America, or the Middle East, where EIMC is widespread by tradition or as a religious ritual, China lacks the history and cultural norms endorsing circumcision. In China, the nation with the largest population in the world, circumcision is generally treated as a selective medical intervention to treat some diseases; only 2.66% of males have been circumcised, and EIMC is not a traditional practice, except among Muslims, who account for < 3% of the population [14].

The culture of circumcision in China is not well understood. The present study is the first to survey parents’ opinions about EIMC in China. In this study, approximately one-third of parents reported that they would agree to circumcise their newborn son; however, the parents were generally not very affirmative and some were not in agreement when making such a decision, suggesting great difficulties in the implementation of EIMC in China. In Chinese traditional culture, it is believed that the body, hair, and skin should not be self-mutilated because they were borne by parents and it is the first step of filial piety. Such traditional beliefs might make people more reluctant to excise a part of the body unless necessary. Although it is difficult to gauge how these traditional perceptions would affect decisions regarding circumcision, such perceptions have probably contributed to the lack of tradition and motivation for circumcision in China. Nonetheless, retention of the prepuce is not regarded as crucial to one’s ethnic identity amongst Chinese; thus, it is likely that traditional beliefs can be changed by promoting education on the health benefits of MC. Modern propaganda, including the internet and television, provides a convenient medium for such education. Furthermore, among those respondents in the current study who considered circumcision for their sons in the future (35.8%), most (74.0%) regarded > 1 year of age to be the best age to undergo circumcision and only 7% thought the neonatal period to be the best time; this confirms the difficulties in implementing EIMC. In a survey from South Korea [18], another Asian country which also lacks a history and tradition of circumcision, it was shown that the circumcision rate is as high as 60% in the entire population and > 90% among high school boys; however, the peak age of circumcision is 9–14 years, and the EIMC rate is also very low [18]. In another study from Zambia, a southern African country with an HIV prevalence of 14% where EIMC has been implemented as a long-term prevention strategy for HIV, it was reported that the acceptability of EIMC is challenged by a fear of negative outcomes, concerns about pain, and issues involving cultural identity [19]. All of these findings, combined with our results, suggest that it will be difficult to implement EIMC in countries without a tradition of circumcision based on culture or religion; conducting awareness campaigns on EIMC will be necessary in such countries to address this issue. Such campaigns could be launched through widespread propaganda via the internet, newspapers, and popular lectures; however, education efforts targeting the parents of newborns by medical workers might be more effective. In the current study, 68% of the respondents commented that medical workers did not give them sufficient information about circumcision, implying that most of the respondents desired to understand EIMC from medical workers in hospitals. This suggests that hospitals might be ideal places to educate the parents of newborn infants while awaiting delivery. This could be achieved by face-to-face education with medical workers or short videos on televisions in the ward. We also observed that the major reason for consenting to EIMC was for health (54.7%), followed by doctor’s advice (31.8%), suggesting that the medical benefits are the primary motivation for EIMC in China. This may be related to an expectation of beneficial effects on health, as suggested by the survey result that 91.0% of respondents considered circumcision to be healthy. The major reason for EIMC unwillingness among parents was pain (50.5%) and the risk (23.5%) of circumcision, which was somewhat different from findings from South Korea, where pain (42.8%) and bad cosmetics (22.5%) were of greatest concern [18]. In fact, EIMC is generally performed under anesthesia worldwide. Combining topical petroleum gel with the dorsal penile nerve block or a subcutaneous ring block is an effective method for controlling post-operative pain, and complications are rare [20]. The surgical complication rate for EIMC is < 1%, with minor bleeding and infection accounting for the majority of cases [16,21]. EIMC is considered to be a cheaper, simpler, and safer procedure compared to circumcision of older boys and men [7,22].

Even so, the fear of pain and the risk of the procedure were reported to be the major barriers to EIMC [9,10,23]. Therefore, EIMC should be done safely to increase acceptance by parents. This can be achieved through proper training, proper instrumentation and sterilization, and complete instructions to the parents of newborns [23]. Devices, such as the AccuCirc, Mogen clamp, and Gomco clamp, which are bloodless, require no sutures, and are associated with minimum pain, are also helpful to ensure the safety of the procedure [21].

Based on this evidence and the fact that most parents (84.2%) would like to know the health benefits of circumcision, as suggested in the present investigation, we speculate that the willingness and acceptance of EIMC would increase in China if the parents of newborns were provided with sufficient information about EIMC (especially the father, who usually has the final say [66.7%]), which is consistent with the findings in reports from other countries [9,19,24]. Indeed, education about EIMC can be easily achieved through free booklets and lectures aimed at the father-to-be. Studies from other countries have shown that providing educational information about the benefits of circumcision and HIV prevention could increase the decision to circumcise a newborn son [25,26].

 One limitation of this study was that we did not investigate the effect of educational interventions on decision-making about EIMC. In addition, the sample of the present study was obtained from Nanjing city, but not from China as a whole, so one should be careful when these findings are generalized to the entire country as regional variations cannot be excluded. Further studies are warranted to evaluate the impact of educational interventions in a more representative sample within China.

## Conclusions

Generally, the willingness and acceptance of EIMC in China is low and the parents of newborns are usually not very affirmative when making a decision regarding EIMC. The major reason for EIMC unwillingness is concern about pain and risk of the procedure. Sufficient education about the benefits of circumcision and HIV prevention is needed if EIMC is to be implemented as an HIV prevention strategy in China.

## Competing interests

The authors declare that they have no competing interests.

## Authors’contributions

Pan Lianjun and Wang Zhong designed the study. Zhang Aixia and Shen Rong conducted the study and collected the data. Pan Lianjun analyzed the data, performed the statistical analysis, drafted the article, and revised the manuscript critically. All authors read and approved the final manuscript.

## Pre-publication history

The pre-publication history for this paper can be accessed here:

http://www.biomedcentral.com/1471-2458/12/738/prepub
